# Videoconferencing Psychotherapy for Panic Disorder and Agoraphobia: Outcome and Treatment Processes From a Non-randomized Non-inferiority Trial

**DOI:** 10.3389/fpsyg.2020.02164

**Published:** 2020-08-21

**Authors:** Stéphane Bouchard, Micheline Allard, Geneviève Robillard, Stéphanie Dumoulin, Tanya Guitard, Claudie Loranger, Isabelle Green-Demers, André Marchand, Patrice Renaud, Louis-Georges Cournoyer, Giulia Corno

**Affiliations:** ^1^Cyberpsychology Lab, Université du Québec en Outaouais, Gatineau, QC, Canada; ^2^Centre Intégré de Santé et de Services Sociaux de l’Outaouais, Gatineau, QC, Canada; ^3^Department of Psychology, Université du Québec à Montréal, Montreal, QC, Canada; ^4^School of Criminology, Université de Montréal, Montreal, QC, Canada

**Keywords:** telepsychotherapy, telehealth, videoconference psychotherapy, panic disorder and agoraphobia, working alliance, self-efficacy, treatment outcome, treatment processes

## Abstract

**Background:**

In the context of the COVID-19 pandemic, legislations are being modified around the world to allow patients to receive mental health services through telehealth. Unfortunately, there are no large clinical trial available to reliably document the efficacy of delivering videoconferencing psychotherapy (VCP) for people with panic disorder and agoraphobia (PDA) and whether basic psychotherapeutic processes are altered.

**Methods:**

This 2-arm intent-to-treat non-inferiority study reports on a clinical trial on VCP and documents how therapeutic working alliance and motivation toward psychotherapy are associated to treatment outcome. We hypothesized that VCP would not be inferior to standard face-to-face (FF) cognitive behavior therapy for PDA. No specific hypothesis was stated to address working alliance and treatment mechanisms. VCP was compared to a gold-standard psychotherapy treatment for PDA, which was delivered either in person or in videoconference, with a strict tolerance criterion of about 2 points on the primary outcome measure. Seventy one adult patients were recruited. Measures of working alliance were collected after the first, fifth, and last session. Motivation toward therapy at pre-treatment and working alliance after the fifth therapy session were used as predictors of treatment outcome and compared with change in dysfunctional beliefs toward bodily sensations.

**Results:**

Panic disorder, agoraphobia, fear of sensations and depressed mood all showed significant improvements and large effect-sizes from pre to post-treatment. Gains were maintained at follow-up. No significant differences were found between VCP and FF, and effect sizes were trivial for three of the four outcome measures. Non-inferiority tests confirmed that VCP was no less effective than FF therapy on the primary outcome measure and two of the three secondary outcome measures. Working alliance was very strong in VCP and did not statistically differ from FF. Working alliance and motivation did not predict treatment outcome, which was significantly predicted by the reduction in dysfunctional beliefs. The strength of the therapeutic bond was correlated with change in dysfunctional beliefs.

**Conclusion:**

Mental health professionals can use VCP to provide services to patients with PDA. Building and maintaining a sound working alliance should not be a source concern. Practical recommendations are formulated.

**ISRCTN Trial Registration Number:**

ISRCTN76456442.

## Introduction

Telemedicine and telepsychotherapy have long been considered solutions to provide health services to people living in rural areas, but legislations are now being modified around the world to allow people to receive services from home due to measures implemented to face the COVID-19 pandemic. However, many people are sensitive to these measures, including some anxious patients and those fearful of physical distancing and confinement. In this context, there is a need for accessible empirical evidences about the efficacy and predictors of outcome of telehealth for each specific mental disorder.

People suffering from PDA are vulnerable in a pandemic crisis, such as the one associated with COVID-19. By definition (American Psychiatric Association [APA], 2013), people with PDA experience recurrent unexpected and spontaneous panic attacks, worry about recurring attacks, and fear of physical symptoms, such as chest pain, heart palpitations, shortness of breath, dizziness, or abdominal distress. PDA is accompanied with significant anxiety about being in places or situations in which it would be difficult to escape or receive assistance if panic attacks were to occur (American Psychiatric Association [APA], 2013), including being confined. The lifetime prevalence of PDA is estimated at 4–6% of the adult population. PDA is chronic, associated with very significant emotional distress, significant fear of body sensations and frequent medical visits (Barsky et al., 1999; Teismann et al., 2018; Chang et al., 2019). The psychological processes at the core of PDA rest on the dysfunctional association between body sensations (interoceptive cues) experienced during panic arousal and perceived threat, which is maintain by avoidance of stimuli or places that elicit feared body sensations or potential panic attacks (Clark, 1986; Barlow, 1988; Taylor et al., 2007). Preliminary reports have suggested that COVID-19 may have an impact on panic disorder (Bhatia et al., 2020; Qiu et al., 2020). People with PDA, or at risk of developing PDA, may be more sensitive to the apprehension of suffering from harmful diseases, experiencing symptoms associated with COVID-19 (e.g., shortness of breath, dizziness), wearing facial masks that may induce the feeling that breathing is difficult, being restricted in mobility because of rules for confinement and physical distancing, having panic attacks induced by the increase in arousal caused by adapting to this situation or by co-morbid anxiety disorders, etc. As an effective treatment for PDA, CBT involves strategies targeting dysfunctional beliefs and avoidance behaviors (Sánchez-Meca et al., 2010). The key treatment mechanism of CBT for PDA is considered to be reappraisal of interoceptive sensations, and to some extent increase in self-efficacy (Clark, 1986; Barlow, 1988; Bouchard et al., 2007; Smits et al., 2012; Gallagher et al., 2013).

Videoconferencing psychotherapy (VCP) is one of the various telehealth modalities that can improve access to mental health professionals trained in evidence-based strategies such as cognitive-behavior therapy (CBT) or with other specialized expertise (Nelson and Duncan, 2015; Liu et al., 2020). The efficacy of CBT is well established in the treatment of anxiety disorders when delivered face-to-face, when compared to no treatment or to a placebo (Hofmann et al., 2012; Carpenter et al., 2018), and is recommended as the gold-standard form of psychotherapy for PDA in clinical guidelines (e.g., Katzman et al., 2014). Several outcome studies have been conducted on VCP, but systematic reviews on anxiety disorders have always concluded that more rigorous research is needed (Rees and Maclaine, 2015; Berryhill et al., 2019).

The most recent systematic review (Berryhill et al., 2019) demonstrated that studies on VCP for panic disorder and agoraphobia (PDA) are scarce. Only three studies have been published so far (Bouchard et al., 2000, 2004; Cowain, 2001; Lindner et al., 2014) and are of moderate methodological quality. One additional study has been published, only in French, not indexed in major databases, and before the entire study was completed (Allard et al., 2007). The largest outcome study on PDA (Bouchard et al., 2004) reported in reviews and meta-analyses (Rees and Maclaine, 2015; Berryhill et al., 2019) was conducted with 21 participants, and showed that CBT delivered by videoconference was effective.

If mental health professionals are to conduct VCP for PDA, it is urgent to share knowledge that demonstrate its efficacy based on larger samples that includes follow-up data. It is also essential to better understand the processes involved in telepsychotherapy, such as the role of working alliance and motivation toward therapy.

Indeed, working alliance is an important part of any psychotherapy and involves three factors: agreement on in-sessions tasks, agreement on treatment goals, and the development of a mutual therapeutic bond (Bordin, 1979; Horvath and Greenberg, 1989). In a systematic review on VCP, Backhaus et al. (2012) found that only 16 out of 47 studies examined the patient-provider relationship in therapy, and 14 out of 16 concluded that patients and providers perceived a strong working alliance. However, a more recent review using different criteria (Norwood et al., 2018) highlighted the need for more studies, including for PDA, and considered that the working alliance was slightly lower in VCP than in FF therapy. Psychotherapists may be apprehensive toward using videoconferencing for fear of disrupting the working alliance (Rees and Stone, 2005; Richardson et al., 2009; Connolly et al., 2020). Two remaining key questions are how the three factors that contribute to working alliance could be affected by VCP and how, in turn, alliance influences treatment mechanisms and outcome.

Another important process that can affect therapy is patients’ motivation. Motivation influences how patients engage in therapeutic work, integrate learning, change their behavior (Deci and Ryan, 2000), and can influence treatment outcome (Orlinsky et al., 1994). Ryan and Deci (2008) proposed that, when individuals are more autonomously engaged in a therapeutic undertaking, they are more likely to integrate learning and to change their behavior, resulting in more positive outcomes. However, to the authors’ knowledge, no study has examined if motivation toward psychotherapy differs when offered in VCP versus face-to-face.

The aims of this paper are to disseminate results on a non-inferiority trial of VCP at post-treatment and follow-up and document factors associated with treatment outcome for PDA. The main hypothesis of the first aim was that VCP would not be inferior to standard face-to-face CBT for PDA according to the primary measure of outcome (severity of PDA). Similar hypotheses were formulated for the three broader measures of generalization (agoraphobic avoidance, fear of sensations and depressed mood). Non-inferiority was defined by a strict and small margin of tolerance for non-inferiority. The second aim was to document the impact of VCP on alliance and how alliance and motivation influenced treatment outcome. No *a priori* hypothesis was stated. First, we compared measures of alliance at the beginning of the treatment, after the first third of the treatment, and the end of the treatment. Second, we assessed and compared the contribution of alliance, motivation, and cognitive changes in dysfunctional beliefs toward body sensations to the primary measure of treatment outcome.

## Materials and Methods

### Procures to Meet Standards in Ethics and Research

The project was approved by the research ethics boards of the lead university and all hospitals involved and was conducted following the ethical standards of the Canadian Tri-Council policy statement for ethical conduct for research involving humans and the Declaration of Helsinki. No monetary compensation was provided. All patients were fully informed of the nature of the study and provided free written consent.

This article was written following CONSORT (Consolidated Standards of Reporting Trials) guidelines for trials assessing non-pharmacological treatments and for non-equivalence trials. There was no modification to the trial’s methods once the study started. Modifications from the grant proposal application were done to respect the budget, ensure feasibility and take into account requests from the ethics committees. Patients and therapists were aware (not blind) of the assigned treatments and study objectives due to the explicit nature of the treatment provided (VCP or FF). The clinical trial was designed as a within-between trial (i.e., pre/post/f-up comparing VCP to FF) without random assignment of participants to the treatment modalities. Random assignment in VCP studies has mixed pros and cons that must be considered. If a study is to replicate the factual and subjective effects due to patients being in a remote location isolated from their therapist, randomly assigning patients to meet online a therapist that is nearby in an adjacent room of the clinic is not an ecologically valid option. This is especially relevant for CBT of PDA, as patients feel reassured by the presence of the therapist during exposure. To use a randomly controlled design, the alternative is to allocate participants to both conditions and, for those in the FF treatment modality, to either have the participants or the therapists commute to the FF therapy site. This solution entails enormous research costs and challenging funding issues. In addition to reducing the representativeness of the study, this solution also significantly increases the risk of drop-out, as experienced by Mitchell et al. (2008) in their study, with a drop-our rate of 40% during therapy. Finally, because remote rural communities are less populated, this approach precludes the recruitment of a large sample. For this study, in order to maximize generalization of results to patients who are unable to receive psychotherapy in FF, participants from a rural (Maniwaki) and an urban (Montréal) distant sites were all allocated to VCP and patients in the local urban site (Gatineau) were all allocated to FF. As per the grant proposal, the study was stopped when funding was exhausted.

Conducting non-inferiority trials is associated with important methodological requirements that must be explicitly stated and justified (Powers and Fleming, 2013; Mauri and D’Agostino, 2017), such as the choice of the reference treatment (to ensure the experimental treatment is not compared with a barely effective one), the selection of the non-inferiority margin, the statistical approach, and the use of an intent-to-treat approach that does no impede the effectiveness of the reference treatment. In the current study, the reference treatment was a gold standard for PDA that has shown its efficacy and superiority over placebo and several other alternatives (Hofmann et al., 2012; Carpenter et al., 2018), and that has been successfully used before by our research group in its traditional FF format (Bouchard et al., 1996). Non-inferiority was defined by a small margin of tolerance operationalized as a Cohen *D* of 0.20, which represents a difference in change between the two conditions of no more than 2 points on the primary outcome measure. The same criterion was applied to the secondary outcome measures. The statistical approach was to document treatment outcome based on repeated-measures ANOVAs, focus on the effect sizes of both conditions’ outcome and the Condition by Time interactions, and interpret the differences in effectiveness based on non-inferiority analyses. Structural equation modeling of latent growth curve model was not use because it requires very large sample size as well as numerous measurement points, and to allow for consistency with the non-inferiority testing approach described above. The trial was analyzed with intent-to-treat design because it is the most conservative approach.

### Sample

**FIGURE 1:**
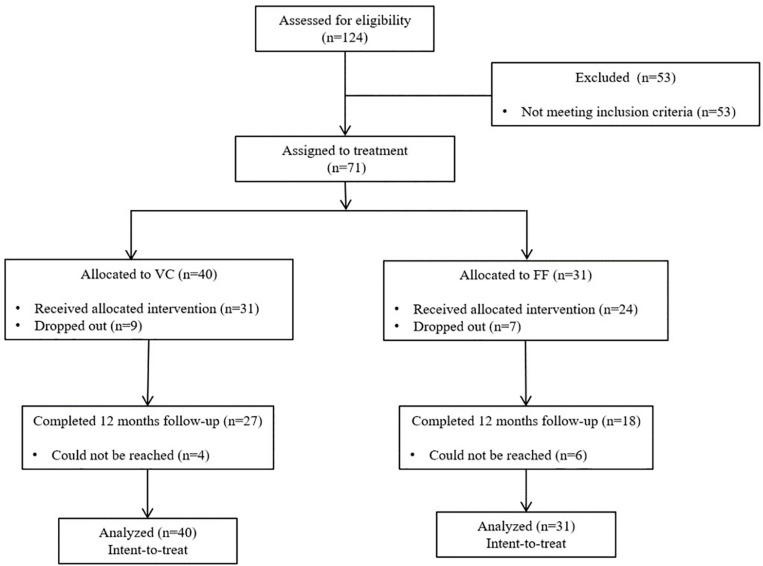
CONSORT flowchart of the progress of participants through the phases of the trial.

Upon contact following publicity and medical references, each participant received the Structured Clinical Interview for DSM-IV (SCID; First et al., 1997) to ascertain eligibility (presence of PDA and other mental disorders). The intake interview was realized face-to-face. The exclusion criteria were: (1) primary diagnosis other than PDA (American Psychiatric Association [APA], 2013); (2) duration of illness of less than 6 months; (3) diagnosis of bipolar disorder, schizophrenia or psychotic disorder, organic mental disorder, intellectual disability, substance use disorder, or severe personality disorders; (4) below 18 or above 65 years of age; (5) currently receiving a psychological treatment (i.e., no concurrent psychotherapy allowed); (6) presence of a medical condition precluding participation in the treatment for methodological or clinical reasons (e.g., cardiovascular disease, Meuniere syndrome, asthma, history of seizures, uncontrolled hypoglycemia, pheochromocytoma, hyper- or hypothyroidism, and brain or lung tumors); (7) if taking antidepressants, using them for less than 6 months or, if taking benzodiazepines, using them for less than 3 months. People on medication who corresponded to the selection criteria were included only if they agreed not to change their medication or to increase its dosage during the study. The vast majority of candidate excluded at the recruitment stage (see Figure 1 for the CONSORT flow chart) were not eligible because PDA was not their principal diagnosis.

The sample size and power were established *a priori* based on results from a previous and separate study (Bouchard et al., 2004) and 124 participants were initially recruited (Figure 1). After intake, the sample consisted of 71 adults who met the selection criteria. Participants from the remote sites were all allocated to VCP (*n* = 40) and patients from the local site all received face-to-face (FF) treatment (*n* = 31). A chi-square analysis was conducted in order to identify differences in dropout rates between VCP and FF and the result was not significant [χ^2^(1) = 0.06, ns].

### Treatment

Treatment consisted of 12 weekly 60-min sessions of CBT and was delivered according to a standardized treatment manual (Clark and Salkovskis, 1987; Barlow and Cerny, 1998; Bouchard et al., 2004; Allard et al., 2007). The treatment was provided without delay, as soon as a participant was deemed eligible for the study. The 12-session written treatment manual was based on target objectives that must all be addressed in a fixed sequence of five modules within a predetermined number of sessions. This allowed some clinical flexibility in treatment pace while protecting fidelity of the delivery of a reproductible validated clinical intervention. The target objectives of the treatment were: building rapport and developing a case formulation (module 1, session 1), sharing a common understanding of information on PDA and the role of appraisal and avoidance of physical sensations (module 1, session 2), conducting cognitive restructuring focused on the core dysfunctional beliefs of PDA as revealed by the case formulation (module 2, sessions 3 and 4), engaging in interoceptive exposure (e.g., hyperventilating, spinning, breathing through a straw) of stimuli and avoidance behaviors identified as relevant in the case formulation (module 3, sessions 5–8), planning and reviewing agoraphobic exposure exercises to be conducted between sessions (module 4, sessions 9–11), and wrapping-up the treatment with relapse prevention (module 5, session 12). The three psychotherapists who conducted all CBT sessions were two female graduate students in clinical psychology and a male psychologist, with previous experience in CBT and trained for the use of VCP. They were weekly supervised by the first author. Treatment integrity (Moncher and Prinz, 1991) was ensured by blind ratings of a subset of video recordings of therapy sessions for adherence to the treatment manual (Allard et al., 2007). Thirty items rated on a 0–3 scale measured therapist’s attitude, general skills, delivery of cognitive restructuring techniques, delivery of exposure, and management of homework assignment. The analysis revealed no difference between the two conditions on the respect of treatment integrity [*t*(1,10) = 1.161, ns]. No adverse effects were reported.

### Equipment and Locations

Two remote cities (Maniwaki and Montreal) were linked at 384 kbps with a local site (Gatineau) with Tandberg 2000 videoconference systems set up in psychologists’ offices. Participants in the VCP condition were all located in the remote sites, treated by therapists located at the local site, and never met their therapist face-to-face. The height of the 32-inch video monitor and the distance between the monitor and the chair were positioned to replicate a face-to-face psychotherapy context. Patient and therapist could see each other from the head to the hips. Therapists in VCP were encouraged to keep the picture-in-picture function activated so they could see their own video image and ensure that they remained visible to their patients. All therapy sessions were video recorded using the videoconference equipment (i.e., using only the camera in the FF condition and turning the monitor off) to assess adherence to the treatment protocol. If documents needed to be shared, email or fax was used.

### Measures

The outcome variables were assessed after the intake diagnostic interview: at pre-treatment, at post-treatment, and at a 12-month follow-up. The duration of the follow-up was set as for 12 months because it is considered as a reasonably long in CBT and by granting agencies [in comparison, in their meta-analysis Carpenter et al. (2018) reported a mean follow-up duration of 5.5 months]. All instruments have been validated and extensively used to assess PDA (see Bouchard et al., 1997 for a review and details of psychometric properties and information). Higher scores represent more severe symptoms. The primary outcome was a measure addressing the severity of PDA globally and was complemented with three secondary outcome measures addressing more broadly the impact of the treatment (agoraphobic avoidance, fear of body sensations, and depressed mood). Additional measures were administered to document predictors of treatment outcome that may be influenced by VCP. They included two variables considered as common factors in all psychotherapies (working alliance and motivation) and two measures specific to CBT of PDA (change in dysfunctional beliefs toward body sensations and in perceived self-efficacy). To maintain an adequate ratio of participants per predictor variables, only change in dysfunctional beliefs toward body sensations were analyzed in the regression analyses reported in the article. The role of perceived self-efficacy was explored in the Online Supplementary Material only. The two measures of working alliance were administered after sessions 1, 5, and 12. All three measurement points were compared to find differences between VCP and FF. To reduce the risks of social desirability biases on measures of working alliance, participants were assured their therapist would not have access to their results; once completed, patients sealed the questionnaires in an envelope and mailed the envelope to the provincial board of psychologists. The envelopes were only returned to the researchers when treatments for all participants were completed. Only ratings of working alliance obtained at session 5 were used in the analyses of the predictors of outcome, as recommended to provide a fair assessment of alliance unbiased by treatment success (Ardito and Rabellino, 2011; Buchholz and Abramowitz, 2020).

#### Panic and Agoraphobia Scale (PAS; Bandelow, 1995)

The PAS was selected as the primary outcome measure because it assesses the global severity of PDA. This self-report has 13 items, rated on a 0 to 4 rating scale measuring: (1) panic attacks (frequency, severity, duration); (2) avoidance; (3) apprehension; (4) impairment in familial and professional relationships; and (5) worries about health. The average score reported for a clinical sample of people with PDA was 24.7 (*SD* = 9.8) and Cronbach’s alpha was 0.88. The PAS is a sensitive and well validated global outcome measure.

#### Mobility Inventory When Alone (MI; Chambless et al., 1985)

This measure of agoraphobia uses 27 items to rate how frequently a person avoids various situations when not accompanied by someone else. Agoraphobic avoidance is a very important feature of PDA and was selected as one of the three secondary measures of the generalization of treatment outcome. The average clinical score reported by the authors was 3.22 (*SD* = 1.01), and an average score of 1.5 (*SD* = 0.45) has been reported for a community sample. The MI-Alone has a Cronbach’s alpha between 0.94 and 0.96.

#### The Body Sensations Questionnaire (BSQ; Chambless et al., 1984)

The BSQ measures the fear of 17 different body sensations and was used as a secondary outcome measure. In the validation study, the average score of the clinical sample was 3.05 (*SD* = 0.85), and an average score of 1.8 (*SD* = 0.59) had been reported in a community sample. The BSQ has a Cronbach’s alpha of 0.87.

#### Beck Depression Inventory (BDI; Beck et al., 1996)

The BDI is a well known 21-item self-report measure of symptoms of depression. As a measure of depressed mood, it is used in several CBT trials to document treatment effects that are broader than core PDA features. The BDI has a Cronbach’s alpha of 0.92. Scores below 10 are in the normal range and scores above 20 are associated with probable or mild depression.

#### The Agoraphobic Cognitions Questionnaire (ACQ; Chambless et al., 1984)

The Agoraphobic Cognitions Questionnaire (ACQ; Chambless et al., 1984) is a well validated measure of the core psychological change processes involved in the CBT of PDA (Clark, 1986). It was administered as a measure of treatment process specific to the CBT of PDA. It consists of 14 items measuring dysfunctional beliefs related to possible catastrophic consequences of having a panic attack. The average score was 2.42 (*SD* = 0.64) in the clinical validation sample, and 1.6 (*SD* = 0.47) in a community sample. The Cronbach alpha is 0.80.

#### Working Alliance Inventory (WAI; Horvath and Greenberg, 1989)

Patients completed the self-rated version of the WAI. This widely used questionnaire measures working alliance with three subscales (agreement on goals, agreement on tasks, and the therapeutic bond). The long 36-item version offers an excellent general measure of working alliance, but it is recommended to analyze the shorter 12-item version if one wants to measure the three first-order unique aspects of the alliance that are the Goal, Task and Bond subscales (Tracey and Kokotovic, 1989). The Cronbach’s alpha are 0.90, 0.90, and 0.92 for the Goal, Task, and Bond subscales, respectively.

#### California Psychotherapy Alliance Scale (CALPAS; Marmar et al., 1986)

The CALPAS is another self-rated measure of alliance. This 24-item instrument was also administered to provide a different and complementary perspective on the working alliance (Bachelor and Salamé, 2000; Buchholz and Abramowitz, 2020).

#### The Client Motivation for Therapy Scale (Pelletier et al., 1997)

The CMOTS was used to provide a global measure patient’s motivation. The 24 items assess assessing intrinsic motivation for therapy, the four forms of extrinsic motivation (integrated, identified, introjected, and external regulation) for therapy, and amotivation for therapy. These factors were derived from Deci and Ryan (2000)’s theory of the self-determination and motivation. This questionnaire was administered at the pre-treatment and the alphas for internal consistency vary between 0.70 and 0.92). The total score was calculated as recommended by the authors and used in this study.

## Results

Data were analyzed using IBM SPSS 25. Table 1 presents the descriptive variables for VCP and FF conditions. Chi-square analyses and Student’s *t*-tests did not reveal pre-existing differences between the two conditions on these variables. Note that there was no statistically significant difference when comparing participants from the different recruitment sites on all of these variables or on outcome variables at pre-treatment.

**TABLE 1:** Descriptive statistics of the sample of participants with panic disorder with agoraphobia who received cognitive behavior therapy.

	VCP (*n* = 40)	FF (*n* = 31)	Statistical test
Age, mean (*SD*)	34.90 (10.45)	36.90 (11.60)	*t*(69) = 0.76, ns
Female	34 (85%)	25 (81%)	χ^2^(1) = 0.24, ns
Presence of at least one comorbid disorder*	19(47%)	19(61%)	χ^2^(1) = 1.34 ns
Canadian	37 (93%)	31 (100%)	χ^2^(1) = 2.43, ns
Education			χ^2^(3) = 7.54, ns
High school (incomplete)	9 (22%)	0 (0%)	
High school completed	10 (25%)	8 (29%)	
College	11 (27%)	7 (29%)	
University	10 (25%)	11(42%)	
Single	20 (50%)	12 (38%)	χ^2^(1) = 0.89, ns
Income			χ^2^(2) = 2.69, ns
Low	14 (35%)	5 (20%)	
Average	18 (45%)	11 (44%)	
High	8 (20%)	9 (36%)	
Motivation toward therapy	13.27 (3.77)	12.3 (4.65)	*t*(67) = 0.96, ns

VCP, videoconference psychotherapy; FF, face-to-face; SD, standard deviation. *Comorbid disorders identified among the sample were: specific phobia (n = 12), generalized anxiety disorder (n = 12), major depressive disorder (n = 9), social anxiety disorder (n = 5), hypochondriasis (n = 3), adjustment disorder (n = 1), obsessive-compulsive disorder (n = 1), insomnia (n = 1), and posttraumatic stress disorder (n = 1).

Repeated measures ANOVAs were performed to document treatment efficacy, and non-inferiority was tested using Wellek (2010) procedures and tables using a strict margin of tolerance for non-inferiority of 0.20 at the significance level of 0.05. All assumptions were respected for the analyses. Mauchly’s test for sphericity was significant and the Greenhouse–Geisser correction was applied. However, the correction was small and yielded the exact same *F* values as when uncorrected.

Table 2 presents results for the PAS, MI, BSQ, and BDI. The ANOVAs revealed significant Time effects for each measure and no significant difference for the Condition and the Condition × Time interactions. Contrasts for Pre to Post Time effects were all significant and very large [for PAS [*F*(1,69) = 79.98, *p* = 0.000, $\eta_{\text{p}}^{2}$ = 0.52), for MI [*F*(1,69) = 43.97, *p* = 0.000, $\eta_{\text{p}}^{2}$ = 0.39], for BSQ [*F*(1,69) = 52.68, *p* = 0.000, $\eta_{\text{p}}^{2}$ = 0.43], and for BDI [*F*(1,69) = 13.99, *p* = 0.000, $\eta_{\text{p}}^{2}$ = 0.17]. Contrasts for Pre to Post by Condition interaction were all non-significant and trivial for all measures, except for the fear of body sensations which was very small [for PAS (*F*(1,69) = 0.2, *p* = 0.63, $\eta_{\text{p}}^{2}$ = 0.003), for MI (*F*(1,69) = 0.08, *p* = 0.78, $\eta_{\text{p}}^{2}$ = 0.001), for BSQ (F(1,69) = 1.65, *p* = 0.2, $\eta_{\text{p}}^{2}$ = 0.023), and for BDI (*F*(1,69) = 0.098, *p* = 0.76, $\eta_{\text{p}}^{2}$ = 0.001)]. Gains were all maintained at the 12-mo follow-up. All posttreatment to follow-up contrasts were non-significant [for PAS (*F*(1,69) = 1.97, *p* = 0.17, $\eta_{\text{p}}^{2}$ = 0.028), for MI (*F*(1,69) = 0.02, *p* = 0.87, $\eta_{\text{p}}^{2}$ = 0.000), for BSQ (*F*(1,69) = 3.32, *p* = 0.07, $\eta_{\text{p}}^{2}$ = 0.046), and for BDI (*F*(1,69) = 0.007, *p* = 0.93, $\eta_{\text{p}}^{2}$ = 0.000)]. Applying Bonferroni corrections with a significance level set at 0.01. did not change the interpretation of the results.

**TABLE 2:** Efficacy of delivering psychotherapy in videoconference or in face-to-face to patients with panic disorder and agoraphobia (with intent-to-treat at post-treatment and follow-up), *N* = 71.

Variable	Condition	Pre		Post		Follow-up		Outcome analysis - ANOVA				Non-inferiority analysis (Tolerance ε = 0.20)	
						
		*M*	*SD*	*M*	*SD*	*M*	*SD*	Time df (2,138)	Condition df (1,69)	Interaction df (2,138)		Pre/post interaction	Pre/F-up interaction
										*F*	Eta squ.	*T*	*T*
PAS	VCP	26.88	9.89	16.43	10.50	15.30	10.82	68.18***	1.52	0.30	0.004	−0.45*	−0.65*
	FF	23.48	8.78	14.06	8.95	13.48	9.72						
MI	VCP	2.89	0.85	2.31	0.88	2.22	0.90	37.31***	1.40	0.449	0.006	0.27*	−0.51*
	FF	2.64	0.99	2.00	0.90	2.07	1.00						
BSQ	VCP	3.08	0.78	2.43	0.78	2.29	0.74	52.35***	0.09	1.10	0.016	1.3	0.72
	FF	3.17	0.78	2.26	0.94	2.29	0.98						
BDI	VCP	12.75	9.31	9.05	7.73	8.70	7.53	11.05***	0.14	0.07	0.001	0.31*	0.01*
	FF	12.41	8.91	8.03	6.93	8.29	7.98						

M, mean; SD, standard deviation; VCP, videoconference psychotherapy; FF, face-to-face; df, degrees of freedom; PAS, Panic and Agoraphobia Scale; MI, Mobility Inventory when Alone; BSQ, Body Sensations Questionnaire; BDI, Beck Depression Inventory. *p < 0.05, **p < 0.01, and ***p < 0.001. Results for the contrasts are reported in the text.

**FIGURE 2:**
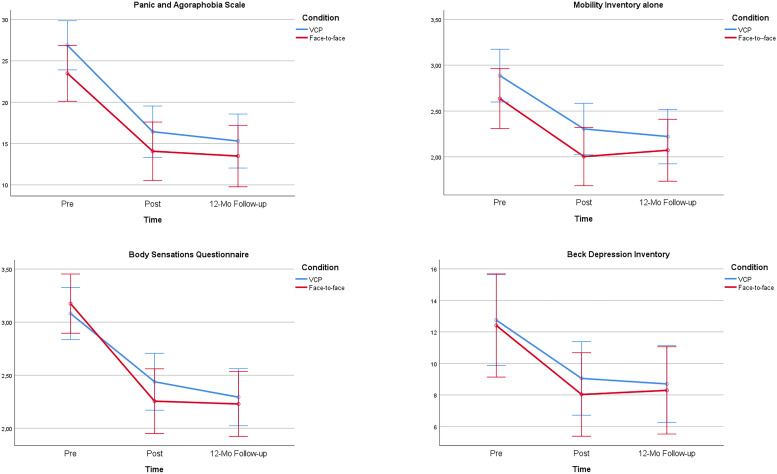
Illustration of 95% confidence intervals for the efficacy of delivering cognitive-behavior therapy to patients with panic disorder and agoraphobia in videoconference psychotherapy (VCP) or in face-to-face.

The analyses were repeated for gender (17% were males) and for presence of none versus at least one comorbid disorder (46% did not report a comorbid disorder) to document the potential impact of these variables. Some impact of gender was found to be statistically significant on three outcome variables, but gender did not significantly influence the impact of VCP on treatment outcome on any variable. For the PAS, the Time X Gender interaction was significant [*F*(2,134) = 5.1, *p* < 0.01, $\eta_{\text{p}}^{2}$ = 0.07], suggesting that males benefited more from CBT than females. For the MI, the main effect of Gender was significant [*F*(1,67) = 10.25, *p* = 0.002, $\eta_{\text{p}}^{2}$ = 0.13], suggesting more severe avoidance in females overall. A similar gender difference was found on the BSQ [*F*(1,67) = 10.1, *p* = 0.002, $\eta_{\text{p}}^{2}$ = 0.13]. The impact of Comorbidity was not statistically significant for any outcome measure. In sum, the treatment was effective, and no difference was found between VCP and FF. Figure 2 illustrates the pattern of results with 95% confidence intervals.

The non-inferiority tests revealed that VCP was statistically no less effective than FF on the primary outcome variable (see Table 2), and two of the three secondary outcome measures (agoraphobia and depressed mood). However, the non-inferiority test did not reach statistical significance for the fear of body sensations.

Repeated measures ANOVAs were also conducted for the measures of working alliance (see Table 3 for the results with patients as treated). A significant Time effect was found with each measure, while no Condition or Time × Condition effects were statistically significant. The quality of working alliance improved during treatment in both conditions and according to both measures. In all comparisons, the alliance was strong but lower in VCP compared to FF, with differences that were not significant and associated with very small effect sizes (partial eta-squared ranged between 0.03 and 0.06). The analyses were repeated with gender and presence of at least one comorbid disorder to document the potential impact of these variables. None of those analyses revealed a statistically significant effect of gender or of presence of comorbidity. Despite the lack of significant main effect for Condition in all ANOVAs, *a posteriori* contrasts were performed to scrutinize the impact of VCP on working alliance. The effect sizes of contrasts comparing VCP and FF were between trivial and small at Session 1 (partial eta-squared of 0.000 for WAI-Task, 0.04 for WAI-Bond, 0.02 for WAI-Goal, and 0.03 for CALPAS), trivial at Session 5 (partial eta-squared of 0.004 for WAI-Task, 0.000 for WAI-Bond, 0.01 for WAI-Goal, and 0.01 for CALPAS), and between trivial and small at Session 12 (partial eta-squared of 0.05 for WAI-Task, 0.04 for WAI-Bond, 0.00 for WAI-Goal, and 0.01 for CALPAS). Further analyses reported in the on-line supplement explored the possibility that a strong working alliance in VCP was obtained because therapists put more efforts than in FF (see Online Supplementary Material). This alternative explanation was not confirmed. The online supplement also reports results of the repeated measures ANOVAs performed with imputed values for missing data on working alliance. These additional analyses did not change the statistical significance of any of the findings pertaining to working alliance.

**TABLE 3:** Strength of the working alliance over the course of psychotherapy delivered in videoconference and in face-to-face and how it relates to treatment outcome for adults with panic disorder with agoraphobia, *N* = 53.

Variable	Condition	Session 1		Session 5		Session 12		ANOVAs			
					
		*M*	*SD*	*M*	*SD*	*M*	*SD*	Time df (2,102)	Condition df (1,51) *F*	Interaction df (2,102)	
								*F*		*F*	$\eta_{\text{p}}^{2}$
WAI-Task	VC	23.94	3.61	25.65	2.70	25.48	3.19	11.39***	1.79	0.39	0.007
	FF	24.68	2.44	26.14	1.70	26.68	1.70				
WAI-Bond	VC	22.16	4.85	24.48	3.53	25.29	3.02	8.09**	2.68	1.88	0.04
	FF	24.64	2.50	24.64	4.85	26.35	2.17				
WAI-Goal	VC	24.84	3.14	26.16	1.71	26.77	1.94	6.99**	3.11	3.19	0.06
	FF	26.52	2.27	26.95	1.43	26.86	1.59				
CALPAS	VC	148.90	14.43	153.60	11.69	155.50	10.51	6.00**	2.48	1.31	0.03
	FF	155.59	9.59	156.41	8.91	158.41	7.08				

**Regression for predictors of residualized improvement on the PAS**											

		***std Beta***	***t***	***sig. p***		***Simple corr.***	***Partial corr.***	***Semi-partial corr.***			

WAI-Task at session 5		−0.04	−0.19	0.85		−0.15	−0.03	−0.03			
WAI-Bond at session 5		−0.21	−1.43	0.16		−0.25	−0.20	−0.2	Statistics for the regression equation		
WAI-Goal at session 5		−0.13	−0.69	0.5		−0.17	−0.1	−0.1	*F*(5,53) = 0.82, *ns*		
CALPAS at session 5		0.09	0.48	0.63		−0.08	0.07	0.07	*R*^2^ = 0.08		
Pre-treatment motivation		−0.08	−0.52	0.61		−0.08	−0.08	−0.07	*Adjusted R*^2^ = −0.17		

*M, mean; SD, standard deviation; VCP, Videoconference Psychotherapy; FF, face-to-face; df, degrees of freedom; WAI, Working Alliance Inventory; CALPAS, California Psychotherapy Alliance Scales; PAS, Panic and Agoraphobia Scale.*

Motivation toward therapy was high and self-determined in participants in the VCP (Mean = 13.26, *SD* = 3.77) and the FF (Mean = 12.30, *SD* = 4.65) conditions. The difference in motivation across conditions at pre-treatment was not significant [*t*(67) = 0.96, *p* = 0.34; $\eta_{\text{p}}^{2}$ = 0.01].

Finally, two regression analyses were performed to identify the predictors of treatment efficacy based on the PAS. The first multiple regression looked at the predictors of outcome with the working alliance (WAI-Task, WAI-Bond, WAI-Goal, CALPAS total score) measured after the fifth therapy session and motivation measured at pre-treatment. Change in pre to post treatment outcome was measured using residualized change score. A second regression was performed to assess the relative role of working alliance and motivation compared to the predictor of change assumed by the CBT model to be the core treatment mechanism, change in dysfunctional beliefs. Residualized change in dysfunctional beliefs were added in the second step of a hierarchical regression, after controlling for the other predictors and residualized change on BDI. Depressed mood was included in the analysis to be more conservative and reduce the percentage of variance left to explained at the second step of the hierarchy (i.e., the impact of change in beliefs was higher when not controlling for the depressed mood). Note that scores on the ACQ significantly decreased following therapy [*F*(2,138) = 41.23, *p* < 0.001; Mean for VCP at pre-treatment = 2.28 (*SD* = 0.59); Mean for VCP at post-treatment = 1.84 (*SD* = 0.47); Mean for VCP at follow-up = 1.75 (*SD* = 0.45); Mean for FF at pre-treatment = 2.48 (*SD* = 0.64); Mean for FF at post-treatment = 1.90 (*SD* = 0.66); Mean for FF at follow-up = 1.88 (*SD* = 0.72)]. The Condition main effect was not significant [*F*(1,69) = 1.23, *p* = 0.27]. The Time by Condition was not significant [*F*(2,138) = 0.047, *p* = 0.63, $\eta_{\text{p}}^{2}$ = 0.007].

The first regression equation was not significant (see Table 3). Result suggested that strength of the working alliance and motivation did not significantly predict treatment outcome. Robustness of our result was assessed by testing *a posteriori* additional regression models. Including the treatment condition in the regression did not change the results. Performing the regression with ratings of the working alliance after the first session, instead of the fifth one, did not change the significance of the regression equation or the predictors, except for agreement on the tasks (*t* = −2.26, *p* < 0.05, semi-partial correlation = −0.29). Using measures of working alliance collected at the last therapy session did not change the significance of the first regression equation or the predictors.

The second regression tested the relative contribution of working alliance, motivation, and the changes in dysfunctional beliefs. After controlling for working alliance (three subscales of the WAI, CALPAS), motivation and change in depressed mood, the addition of residualized change scores on the ACQ lead to a significant regression model [*F*(7,52) = 3.89, *p* < 0.002, *R*^2^ = 0.37, *adjusted R*^2^ = 0.28; *F change* (1,45) = 7.54, *p* = 0.009]. All parameters that were non-significant in the previous regression remained non-significant, change in BDI was significant (std Beta = 0.36, *t* = 2.45, *p* = 0.014, semi-partial correlation = 0.30) but, most importantly, change in dysfunctional beliefs was significant (std Beta = 0.37, *t* = 2.75, *p* = 0.009, semi-partial correlation = 0.32). Of note, the correlation between the bond subscale of the WAI at session five was significantly correlated with change in dysfunctional beliefs (*r* = −0.29, *p* < 0.025), which was not the case for the other measures of alliance and motivation. The online supplement reports results with imputed values for missing data and for self-efficacy. Analyses with imputed values did not change the interpretation of the results, and the role of self-efficacy was found to be statistically significant.

## Discussion

This study provides important information to guide the delivery of mental health services via teleconference technologies during and after the COVID-19 crisis. Results found no evidence of CBT for PDA being significantly less effective when delivered in VCP compare to FF on all outcome measures. The treatment was effective at post-treatment and gains were maintained at follow-up based on measures of panic disorder, agoraphobia, fear of sensations and depressive mood. Confirming the main hypothesis of the first aim of the study, the non-inferiority analysis demonstrated that VCP was significantly non-inferior to FF therapy for the primary outcome measure of PDA. Two of the secondary hypotheses were also confirmed, showing significant non-inferiority for agoraphobic avoidance and depressed mood. However, one of the secondary hypotheses was not supported for the measure of fear of physical sensations. There was no significant difference in treatment outcome on the fear of sensations, but study lacked sufficient power to reach the significance level of non-inferiority with a strict tolerance criterion. The experimental design retained for the study reflects the situation of patients who are unable to meet the therapist to receive face-to-face care and could not feel reassured by her or his physical proximity during therapy sessions. A gender difference in treatment response, regardless of the treatment modality, was observed. This is likely to be related to the small number of males in the study, which is consistent with the gender distribution of PDA, and the impact of a few strong male responders in each condition.

The second significant finding is that CBT can be conducted in VCP with an excellent working alliance. The use of instruments measuring working alliance from two different theoretical perspectives provides an interesting perspective. The CALPAS has been used less frequently in studies on CBT (Buchholz and Abramowitz, 2020) and provides information that complements the WAI, such as patient working capacity, patient commitment and therapists understanding and involvement. This is reassuring for mental health professionals who may worry that using technology to remotely deliver psychotherapy may pose significant threat to the working alliance and the therapeutic relationship (e.g., Rees and Stone, 2005). Motivation at pre-treatment was also not a source of concern. Working alliance, when measured globally with the CALPAS and at the specific component level with the subscales of the WAI, was not a significant predictor of outcome, which is consistent with other studies on CBT for PDA (see Buchholz and Abramowitz, 2020 for a more elaborated discussion). Consistent with the CBT model, the key factor associated with treatment outcome was change in dysfunctional beliefs. Change in beliefs was correlated with the possibility to build a strong bond with the therapist at session 5, even when therapy was delivered remotely. This is clinical meaningful, as it supports the notion that: (a) a strong alliance can be built in VCP, including the development of a strong therapeutic bond, (b) a strong bond is necessary in CBT to engage in the key behavioral techniques that lead to cognitive change, which (c) is the key factor leading to improvement and treatment success.

Our results confirm with a larger sample and methodological improvements the efficacy of delivering CBT in VCP for PDA (Bouchard et al., 2000, 2004; Cowain, 2001; Allard et al., 2007; Lindner et al., 2014). In addition, they contribute to the growing body of evidence that using videoconference does not significantly compromise the quality of the three factors of working alliance, or the alliance measured globally (Bouchard et al., 2004; Allard et al., 2007; Germain et al., 2010; Backhaus et al., 2012). Motivation before initiating therapy was slightly higher in VCP participants, but this was not significant and did not influence treatment outcome. Our study used a global motivation score, and it would be worthwhile to examine the role of individual motivation subtypes in future studies. When considering whether or not using VCP, some professionals and patients may have experienced low levels of telepresence in their professional or social use of videoconference. In VCP, telepresence refers to the impression of really being *in* therapy with the provider, rather than being in a physically different location (Bouchard et al., 2011). The feeling of telepresence in VCP could have an impact on the quality of working alliance, especially on the bond between patient and therapist, and may indirectly influence treatment outcome. Telepresence (Draper et al., 1998) is expected to differ when comparing psychotherapy to common applications of videoconference, such as business meetings, classes and social events. An experimental study (Bouchard et al., 2011) showed that videoconference exchanges involving emotions, akin to those observed in psychotherapy, compared to more neutral ones, led to stronger telepresence. More research on the role of telepresence and working alliance on psychotherapy processes is required. However, in the meantime, some tentative suggestions can be formulated to build and manage a working alliance using e-mental health delivery methods during the COVID-19 pandemic. First, therapists must focus more on the general felling of telepresence occurring during the session than on small communication glitches that can occur during VCP. Second, it is important to be aware and address explicitly breaks in acceptance of the technology settings (Haddouk et al., 2018; situations when patients become frustrated toward the use of VCP). Third, therapist may want to look directly at the camera to establish direct eye contact with their patient, instead of looking at the eyes of the patient on the video monitor. Fourth, therapist may need to use more non-verbal cues (e.g., nodding or thumbs up) and allow longer pauses between verbal exchanges with their patient to reduce the risks of talking over each other. Finally, therapists can explore the literature on ways to communicate empathy in computer-mediated interactions (Grondin et al., 2019). More research is also required to extend our results to other mental disorders, including those for which building and maintaining a strong working alliance is more challenging than for anxiety disorders (e.g., addictions, personality disorders).

The study has limitations that must be acknowledged. First, participants were not randomly assigned to both conditions, for practical reasons that allowed to replicate situations where patients are remote and isolated from their therapist. Conducting exposure to interoceptive cues in a context where the patient is far away from the therapist is an important asset for the generalization of the current study to the situation imposed by the COVID-19 and public health rules related to physical distancing and confinement. Actually, most past VCP studies did not conduct randomized control trials (Berryhill et al., 2019). The 12-month follow-up must be interpreted in the context of an intent-to-treat analysis where some patients could not be reached to collect information. Finally, participants were aware they would receive VCP when they volunteered for the study. Volunteers for the study may thus have had a more positive attitude toward VCP than the general population. However, in situations where telemedicine is a viable solution, or the only solution, the impact of attitude toward technology may be less important than actually having access to services.

In the light of our results, three clinical issues deserve comments regarding the application VCP for PDA in the context of COVID-19: (a) fear of the disease, (b) confinement, and (c) deconfinement, physical distancing and other public health measures. Dysfunctional thoughts and beliefs about diseases, health conditions or treatments, can be addressed effectively in VCP by cognitive restructuring techniques and exposure to interoceptive cues. In the current study, all CBT interventions were based on an individualized case conceptualization. In the context of COVID-19, it would be important to consider exploring with patients if the virus, the disease, the potential treatments (including intubation), the potential vaccines, information from the Internet and peers, or the rules imposed by public health services, contribute to PDA (e.g., Bhatia et al., 2020). Cognitive restructuring and exposure should be adapted accordingly. Some patients may avoid going to hospitals and clinics to receive relevant physical care or exams by fear of contracting the virus. Therapists must also pay attention to subtle avoidance behaviors that may be hidden under good intentions (e.g., staying home may be recommended as a preventive measure, but it may also be a justification for not wearing a facial mask and venture outside). Confinement imposed by public health authorities, or self-imposed by house bound PDA patients, can be a sound justification for opting for VCP. The current study shows that it is an excellent solution and illustrates that interoceptive exposure is feasible in VCP, including hyperventilation, breathing through a straw, doing aerobic exercises, spinning, Valsalva maneuver, etc. (Clark and Salkovskis, 1987; Barlow and Cerny, 1998). The therapeutic bond was excellent in the current study when these exposure exercises were introduced, and it remained high until the end of a treatment that relied heavily on exposure. However, at some point, VCP must encourage patients with agoraphobia to actively leave the comfort of locations where they feel safe and reassured. With smartphones and other communications devices, VCP sessions can even be conducted when patients are exposing themselves in feared locations. Whenever possible, exposure to agoraphobic situations must be targeted and addressed. When not possible, therapists must use alternative strategies (e.g., imaginal exposure, videos, virtual reality) or postpone exposure. However, technology must not become a way to foster avoidance in anxious patients. Finally, measures imposed by public health authorities to cope with COVID-19 are much more diverse than confinement and each of them may impact the clinical management of PDA. For example, wearing facial masks may induce sensations feared by PDA patients (e.g., difficulty breathing). Long lines and queue to access stores and services can be feared and avoided by people with PDA. Physical distancing and other deconfinement rules may limit the techniques the therapist could apply in the office (e.g., hyperventilating is very likely to have a different impact on the spread of respiratory droplets compared to talking 2-m away from each other), and coping with the changes imposed by public health and safety may increase the daily arousal that facilitate the onset of panic attacks in people with PDA. Finally, therapists and patients may want to consider an option that has not yet been explored in clinical trials, which is alternating between VCP and FF every few sessions.

To conclude, additional general practical guidelines for use of VCP are summarized. To start with, not all telehealth services need to use videoconference. Telephone, web-based treatments and other options are worth considering given each patient’s and therapist’s contexts. When it comes to VCP, the selection of the software to use for VCP must be considered carefully. In addition to practical and ergonomic issues, their use must respect the rules and regulations implemented by the regulatory bodies of each country, province, or state. Even in open markets (e.g., European Union, Canada - United States -Mexico Agreement), there are constraints and limitations to the use of titles such as psychotherapist or psychologist, rights to practice psychotherapy, and established best practices to protect confidentiality. Psychotherapy and behavioral change are not limited to the VCP session; for patients it is a process that requires personal engagement, emotional processing, time, perspective taking, and between sessions exercises. For therapists, it also implies using the right software. Some software needs a password to confirm the identity of the patient and restrict access on the users’ computer, offers robust encryption of the therapy session and uses servers that protect confidentiality. In terms of psychotherapeutic context, it remains important at the start to define and agree with patients on the psychotherapeutic frame. For example, setting rules for appropriate physical space on both ends (e.g., privacy, not being disturb while in session), interpersonal interactions (e.g., no emergency calls outside office hours, keep VCP interactions similar to face-to-face), management of distractors (e.g., no email alerts during session), communication strategies (e.g., use of non-verbal interactions to signal approval instead of speaking over each other, connect a few minutes before the session to replicate the experience of settling down in the waiting room), and a contingency management plan if the sessions fails abruptly (e.g., rescheduling versus calling back on the telephone or without video feed). For health care agencies and regulatory board, results of this study should encourage them to guide and inform their mental health professionals on the relevance and potential of VCP. The publication of telehealth guidelines, consent form examples and which software to recommend should be among their list of key priorities during and after the COVID-19 crisis.

## Data Availability Statement

The raw data supporting the conclusions of this article will be made available by the authors, without undue reservation.

## Ethics Statement

The studies involving human participants were reviewed and approved by Comité d’Éthique de la Recherche (CÉR) de l’Université du Québec en Outaouais (UQO). The patients/participants provided their written informed consent to participate in this study.

## Author Contributions

SB had full access to all the data in the study and took responsibility for the integrity of the data and the accuracy of the data analysis. All of the authors of this article provided a significant intellectual contribution to this study, including its conception, the acquisition and analysis of data, drafting the article or providing critically important review. SB was the lead researcher, conceptualized the study and its design, wrote the grant application, supervised the study, conducted the analyses, and finalized the article. MA contributed to the conceptualization of the study, was involved in data collection and her doctoral thesis is based on analyses of a subset of the data. GR contributed to the study design and the coordination of the study. SD contributed to the coordination of the study and complementary analyses. TG contributed to the design and the analysis of the material in the online supplement. CL contributed to the final analyses and wrote the first draft of the article. IG-D, PR, and L-GC contributed to the conceptualization of the study and the original research grant. AM contributed to the conceptualization of the study, the original research grant, and the supervision of the study. GC contributed to the final analyses and critical work on the final versions of the article. All authors contributed to the article and approved the submitted version.

## Conflict of Interest

SB is the president of and owns equity in Cliniques et Développement In Virtuo, a university spin-off that uses virtual reality as part of its clinical services and distributes virtual environments. The terms of this arrangement have been reviewed and approved by the Université du Québec en Outaouais in accordance with its conflict of interest policies. GR is VP of Corporate affairs and owns equity in In Virtuo. The remaining authors declare that the research was conducted in the absence of any commercial or financial relationships that could be construed as a potential conflict of interest.
