# Supplementary material for: Videoconferencing Psychotherapy for Panic Disorder and Agoraphobia: Outcome and Treatment Processes From a Non-randomized Non-inferiority Trial
Source: Front Psychol. 2020 Aug 21;11:2164. doi: 10.3389/fpsyg.2020.02164 (PMC7472915; doi:10.3389/fpsyg.2020.02164)
Supplement: Supplementary file 1 [file Table_1.DOCX]

**Online Supplement material**

**Exploring if therapists are overcompensating in VCP to build a strong working alliance.**

It is plausible that the lack of significant difference in working alliance between VCP and FF could be explained by therapists in VCP investing extra efforts to promote and maintain a strong alliance and compensate for the use of videoconference to communicate. This hypothesis was explored with the data available from the video recordings of the therapy sessions. Picture-in-picture option was enabled during most VCP sessions and displayed the video of the therapist in a window on a corner of the screen displaying the image of the patient. In FF, recordings of the sessions always captured verbal exchanges and the image of patient, but unfortunately the therapists were not always visible (the TV monitor was turned-off so they could not be aware they were not visible on the camera). For all available video recordings where it was possible to see the patient and the therapist, a subset of sessions was randomly picked to be analyzed. We analyzed 63 therapy sessions conducted in VCP and 22 in FF.

Therapist’s behaviors and attitudes were examined by a research assistant blind to the results of the patient’s questionnaires on the outcome and treatment process measures. The Behaviors and Attitudes Enhancing Working Alliance rating scale (BAEWA; Lefebvre and Bouchard, 2005) was developed based on a literature review and items from the Psychotherapy Process Q-set (Jones, 2000) and the Vanderbilt Psychotherapy Process Scale (O’Malley et al., 1983). The research assistant rated 18 items using a Likert scale ranging from 0 (“Not at all”) to 10 (“Completely”).

Paired t-tests were performed, adjusted for non-equal variance when necessary, to compare the ratings obtained for each behavior (see Supplement Table below). To maximize the probabilities of finding significant differences, no Bonferroni correction was applied. This statistical position increases the likelihood of finding false positives, which is preferable in the context of exploring alternative hypotheses that can shed light on the lack of statistical difference on working alliance. The interpretation of significant differences on the BEAWA must be interpreted in the light of this statistical approach and other methodological limitations.

Significant differences were observed in 7 out of 18 therapists’ behaviors and attitudes, and only two may be in the direction of efforts from therapist to compensate to boost the alliance. Among the scores rated as significantly different in VCP which could have a positive impact on the alliance, we found that therapists interrupted and talked over patients *less* frequently and appeared less irritated by patients’ behaviors and attitudes. We attribute the finding that therapists may interrupted less their patients in VCP to their training and experience with VCP, which made them aware that VCP favors talking over each other and can be prevented by respecting longer pauses. The other five significant differences were in favor of behaviors and attitudes that could strengthen the alliance in FF, such as a Socratique attitude, reformulation, putting emotional reactions in perspective, respecting silences, and therapists expressing themselves more clearly. Therefore, we found no convincing evidence to support the possibility that therapists could be actively compensating and engaging in more behaviors that promote and maintain a strong alliance in VCP.

**Exploring the impact of imputing missing data and the role of perceived self-efficacy.**

Randomly missing data have been identified for the measures of motivation (2 missing data), working alliance after the first therapy session (5 missing data), after the fifth therapy session (12 missing data), and after the last therapy session (15 missing data). To explore if replacing missing data would impact the results, additional analyses were conducted with multiple imputations performed using the linear regression method and Mersenne Twister random generator of SPSS v.25. Five sets of imputed values were produced and analyzed by SPSS. Findings reported in Table 3 of the main article were replicated with a complete sample of 71 participants with imputed missing values. None of the statistical differences that were significant in Table 3 of the main article became non-significant, and none of the non-significant differences became significant. The partial eta-squared assessment of effect sizes of main Condition effect (VCP vs FF) estimated with imputed data ranged between .00 and .03 for the Task subscale of the WAI, between .00 to .05 for the Bond subscale, between .00 to .06 for the Goal subscale, and between .00 and .04 for the CALPAS. In all instances, the largest (and not statistically significant) effect sizes were those found with the original non-imputed data reported in the main article.

The first regression analysis from Table 3 in the original article was replicated with the data imputed for missing values. The regression models remained not statistically significant and the adjusted R^2^ in the same range of very low variance explained by the subscales of the WAI and the CALPAS administered after the fifth session and the pre-treatment motivation. The (non-significant) semi-partial correlations for the combined model were -.098, -.16, -.023, .053 and .024 for the Task subscale, the Bond subscale, the Goal subscale, the total score of the CALPAS and the motivation measures, respectively.

The second regression analysis was also revised with a sample of 71 participants with data imputed for missing values for the measures of motivation and working alliance. The increase in sample size allowed for including of the measure of self-efficacy along with the measure of dysfunctional beliefs in the second step of the regression model. Self-efficacy was measured with the Self-Efficacy to Control Panic Attacks Scale (see Bouchard et al., 2004). An increase in self-efficacy express improvement in outcome. After controlling for the working alliance (three subscales of the WAI, CALPAS; with imputed missing values), motivation (with imputed missing values) and change in depressive mood, the addition of residualized change scores on the ACQ and residualized change scores on the SE-CPAQ lead to a significant regression model [F(8,69)=5.15, p < .000, *R^2^*=0.55, *adjusted R^2^* =0.50] explained by the addition of the cognitive variables in the second step of the hierarchical regression [F*change* (2,61)= 14.04, p < .000]. All parameters that were non-significant in the previous regression remained non-significant in the final combined model. Over and above change in depressed mood, which was significant in the first step of this regression, working alliance and motivation, change in dysfunctional beliefs was significant (t=2.75, p = .006, semi-partial correlation = .24). Change in self-efficacy was also statistically significant (t=-2.38, p = .017, semi-partial correlation = -.21). In sum, results of analyses conducted with imputed data support the findings reported in the original article: change in cognitive variables consider by CBT models as core factors leading to improvement in PDA remain the best predictor of treatment outcome in VCP. The larger sample size allowed to also document the role of self-efficacy in VCP of PDA.

**References for online material**

Bouchard, S., Paquin, B., Payeur, R., Allard, M., Rivard, V., Fournier, T., et al. (2004). Delivering cognitive-behavior therapy for panic disorder with agoraphobia in videoconference. Telemed. J. E. Health 10, 13-25. doi: 10.1089/153056204773644535

Jones, E.E., (2000). Therapeutic action: A Guide to Psychoanalytic therapy. N.J. Aronson.

Lefebvre, C. and Bouchard, S. (2005). Développement d’un outil permettant d’évaluer l’impact des comportements et des attitudes du client et du thérapeute sur la qualité de la relation thérapeutique. / Developpement of an instrument devised to assess the impact of client’s and therapist’s attitudes and behaviors on the quality of the therapeutic relationship. Université du Québec en Outaouais. Unpublished honours thesis. Available in French upon request from the corresponding author.

O’Malley, S.S., Suh, C.S., and Strupp, H.H. (1983). The Vanderbilt Psychotherapy Process Scale: A report on the Scale Development and process-outcome Study. J. Consult. Clin. Psychol. 51, 581-586. doi: 10.1037/0022-006X.51.4.581

| **Online Supplement Table.** Exploring potential differences observed in video recording of therapist’s behavior and attitudes that may have influence working alliance when conducting videoconferencing psychotherapy (VCP) of face-to-face (FF) sessions. | | | | | | | | | |
| --- | --- | --- | --- | --- | --- | --- | --- | --- | --- |
|  | **VCP** | | | **FF** | | | df | *t* | *p* |
|  | n | *M* | *SD* | n | *M* | *SD* |  |  |  |
| Therapist supports client and encourages him/her | 63 | 7.08 | 1.126 | 22 | 6.91 | 1.571 | 83 | .549 | .585 |
| Therapist help client to focus on functional thoughts | 63 | 6.79 | 1.628 | 23 | 7.96 | 1.430 | 84 | -3.024 | .003* |
| Therapist uses reformulations | 62 | 2.37 | 1.321 | 22 | 3.27 | 1.695 | 82 | -2.548 | .013* |
| Therapist brings patient to question the nature of his/her reactions to the problem | 63 | 7.16 | 1.428 | 23 | 8.26 | 1.010 | 84 | -3.399 | .001* |
| Therapist respects moments of silence | 62 | 5.19 | 1.595 | 22 | 6.14 | 1.612 | 83 | -2.388 | .019* |
| Therapist lets client express himself at his/her own pace | 63 | 7.87 | .729 | 23 | 8.17 | .717 | 84 | -1.701 | .093 |
| Therapist expresses his/her intentions with non-verbal behaviors | 62 | 6.71 | 1.046 | 23 | 6.13 | 1.632 | 28.9 ª | 1.585 | .124 |
| Therapist encourages client to invest himself/herself in therapy | 63 | 7.48 | .800 | 23 | 7.74 | .964 | 84 | -1.275 | .206 |
| Therapist stays authentic in therapy | 63 | 8.38 | .490 | 23 | 8.48 | .511 | 82 | -.807 | .422 |
| Therapist respects client and accepts him/her | 61 | 8.38 | .610 | 23 | 8.43 | .728 | 82 | -.367 | .715 |
| Therapist reacts to client’s non-verbal behaviors | 63 | 5.40 | 1.582 | 22 | 5.82 | 1.763 | 83 | -1.044 | .299 |
| Therapist express himself/herself clearly | 63 | 8.19 | .396 | 23 | 8.74 | .449 | 84 | -5.487 | .000** |
| Therapist is focused on the task at hand | 63 | 8.35 | .626 | 23 | 8.65 | .775 | 84 | -1.860 | .066 |
| Therapist uses humour | 63 | 2.41 | 1.444 | 23 | 2.57 | 1.376 | 84 | -.439 | .662 |
| Therapist interrupts the client | 63 | 1.52 | .780 | 23 | 2.26 | .964 | 84 | -3.636 | .000** |
| Therapist's conversation is focused on precise goals | 63 | 8.25 | .761 | 23 | 8.48 | .846 | 84 | -1.174 | .244 |
| Therapist appears irritated by client’s behaviors or attitudes | 63 | 1.02 | .833 | 23 | 2.35 | 2.228 | 24.28 ª | -2.797 | .010* |
| Therapist is optimistic about therapy | 63 | 7.65 | .699 | 23 | 7.22 | 1.413 | 26.04 ª | 1.410 | .171 |

*Note.* M=Mean; SD=Standard deviation; VCP=Videoconferencing Psychotherapy; FF= Face-to-face psychotherapy; df=degrees of freedom; * *p* < .05; ** *p* < .001; ª degrees of freedom adjusted for unequal variance when the Levene test was significant.
